# The role of the gut microbiome in graft fibrosis after pediatric liver transplantation

**DOI:** 10.1007/s00439-020-02221-8

**Published:** 2020-09-13

**Authors:** Tian Qin, Jingyuan Fu, Henkjan J. Verkade

**Affiliations:** 1 Pediatric Gastroenterology/Hepatology, Section of Nutrition and Metabolism, Research Laboratory of Pediatrics, Department of Pediatrics, Beatrix Children’s Hospital/University Medical Center Groningen, P.O. Box 30.001, 9700 RB Groningen, The Netherlands; 2 Department of Genetics, University of Groningen, University Medical Center Groningen, Hanzeplein 1, 9713 GZ Groningen, The Netherlands

## Abstract

Liver transplantation (LT) is a life-saving option for children with end-stage liver disease. However, about 50% of patients develop graft fibrosis in 1 year after LT, with normal liver function. Graft fibrosis may progress to cirrhosis, resulting in graft dysfunction and ultimately the need for re-transplantation. Previous studies have identified various risk factors for the post-LT fibrogenesis, however, to date, neither of the factors seems to fully explain the cause of graft fibrosis. Recently, evidence has accumulated on the important role of the gut microbiome in outcomes after solid organ transplantation. As an altered microbiome is present in pediatric patients with end-stage liver diseases, we hypothesize that the persisting alterations in microbial composition or function contribute to the development of graft fibrosis, for example by bacteria translocation due to increased intestinal permeability, imbalanced bile acids metabolism, and/or decreased production of short-chain fatty acids (SCFAs). Subsequently, an immune response can be activated in the graft, together with the stimulation of fibrogenesis. Here we review current knowledge about the potential mechanisms by which alterations in microbial composition or function may lead to graft fibrosis in pediatric LT and we provide prospective views on the efficacy of gut microbiome manipulation as a therapeutic target to alleviate the graft fibrosis and to improve long-term survival after LT.

## Introduction

Pediatric liver transplantation (LT) has become a standard procedure for children with end-stage liver disease, for example due to biliary atresia or progressive familial intrahepatic cholestasis. The number of LTs performed globally has been reported to be 4 to 9 per million population younger than 18 years, with a 10-year survival rate higher than 80% (Bourdeaux et al. 2009; Fischler et al. 2019).

Notwithstanding the high survival rate of LTs, up to 50% of pediatric LT patients develop graft fibrosis in 1 year after transplantation, based on protocol biopsies. (Evans et al. 2006; Scheenstra et al. 2009). Liver fibrosis is a well-known consequence of chronic liver injury occurring in a variety of liver diseases, including genetic diseases, hepatitis, and metabolic diseases. Fibrosis is pathophysiologically considered as a wound healing response in reaction to repeated liver injury, leading to progressive accumulation of extracellular matrix (ECM). Interestingly, pediatric patients with post-transplant graft fibrosis frequently do not have clear indications of graft complications or of (prior or ongoing) elevations in liver biochemistry, in contrast to conditions leading to liver fibrosis before LT. Thereby, graft fibrosis can be considered as a “silent fibrosis”. The clinical consequence of graft fibrosis is not well known, but 29% of patients with graft fibrosis can progress to cirrhosis, which may result in graft dysfunction and ultimately the need for re-transplantation (Scheenstra et al. 2009). Therefore, understanding the underlying risk factors of graft fibrosis is clinically important.

Many factors have epidemiologically been associated with graft fibrosis, including donor age, prolonged ischemia time, transplant‐related or immune-related factors such as biliary/vascular complications, subclinical rejection, and post-transplantation lymphoproliferative diseases (PTLD) (Chanpong et al. 2019; Rhu et al. 2020; Scheenstra et al. 2009; Tokodai et al. 2018; Ueno et al. 2016). However, these factors do not provide a satisfactory explanation for the high incidence of graft fibrosis nor the underlying mechanisms.

The intestinal microbiota in a human adult consists of 10^13^–10^14^ microorganisms and has been shown to play an active role in many aspects of health and disease (Lynch and Pedersen 2016; Sender et al. 2016; Valdes et al. 2018). Microbial dysbiosis refers to an “imbalance” in the gut microbial community that is associated with diseases, which is often characterized as lower diversity, an increase of potentially pathogenic taxa, and a decrease of beneficial taxa when compared to a healthy microbiota. The crosstalk between the gut and liver is increasingly being recognized (Albillos et al. 2020; Tripathi et al. 2018). Microbial dysbiosis could be complicit in liver disease progression and has long been associated with various liver diseases, such as nonalcoholic fatty liver disease (NAFLD) (Boursier et al. 2016; Schwimmer et al. 2019; Zhu et al. 2013), alcoholic liver disease (ALD) (Bajaj 2019), cirrhosis and its complications (Chen et al. 2011). Animal studies have demonstrated the transferrable phenotype of NAFLD (Yuan et al. 2019) and ALD (Llopis et al. 2016) via the transplantation of disease-associated fecal microbiota (fecal microbiota transplant, FMT). Reversely, modulation of the microbiota could help ameliorate liver injury (Dhiman et al. 2014; Liu et al. 2019). These observations support a causal contribution of microbiota to the pathogenesis of liver diseases. Furthermore, the gut microbiome has been reported to influence the success rate of solid organ transplantation, including renal and intestinal transplantation (Ardalan and Vahed 2017; Chenyang Wang 2018), and hematopoietic stem cell transplantation (Peled et al. 2020).

From a perspective view, we herewith hypothesize that the gut microbiome is an important determinant for the development of liver graft fibrosis and that restoration/manipulation of the gut microbiome after pediatric LT can mitigate or even prevent post-LT graft fibrosis. Microbial dysbiosis is already present in pediatric patients with end-stage liver diseases. Due to the cross-talk along the gut-liver axis, we expect a feed-back regulation between the gut microbiota and the liver. After LT, altered microbial composition and/or function can be ameliorated or even reversed due to the restoration of liver function. In turn, balanced gut microbiome is essential to maintain normal liver function. However, the microbial composition/function may not be fully restored after LT. If alterations persist post-transplantation, we hypothesize that they contribute to the development of graft fibrosis. This review aims to provide an overview of graft fibrosis after pediatric LT, to summarize the current-of-state discoveries of the gut microbial associations before and after LT, to discuss potential mechanisms underlying the role of the gut microbiome in the development of graft fibrosis, and to suggest microbiome-targeted approaches to alleviate fibrosis. Finally, we propose an experimental framework to explore this perspective.

### Graft fibrosis after pediatric LT

According to the primary location in the hepatic acinus, graft fibrosis has been differentiated into portal, sinusoidal and centrilobular fibrosis (Venturi et al. 2014). The different locations of fibrosis have been associated with different biochemical profiles and risk factors. Portal fibrosis has been correlated with abnormal liver function, prolonged ischemia time, deceased donor grafts, and a history of PTLD or rejection while sinusoidal fibrosis was related to biliary complications and abnormal liver function (Baas et al. 2017; Rhu et al. 2020; Venturi et al. 2014). Centrilobular fibrosis was associated with vascular complications, presence of autoantibodies, gamma-globulins levels, donor factors, and history of PTLD (Rhu et al. 2020; Venturi et al. 2014). Autoantibodies are immunoglobulins that recognize host antigens and commonly associate with chronic liver injury. The presence of centrilobular inflammation and fibrosis therefore would suggest some form of chronic rejection in the allograft (Egawa et al. 2012; Hassoun et al. 2004; Sundaram et al. 2006). Baas et al. (2017) confirmed the high prevalence of fibrosis post-LT and reported associations of the three acinar locations of fibrosis with different clinical variables, indicating different mechanisms involving in graft fibrosis. For example, chronic, low-grade rejection could be involved in the development of graft fibrosis (Feng et al. 2018; Yamada et al. 2012). Graft rejection is mediated via pathways of allorecognition, the processing and presentation of donor antigens to recipient cells. Especially, chronic rejection can contribute to graft injury via antibody-mediated or T cell-mediated mechanisms (Lee 2017; Tedesco and Grakoui 2018).

Several observations do support the contribution of antibody-mediated immunity processes to the development of fibrosis. For example, donor-specific antibodies (DSA), either present before transplantation or de novo generated after transplantation, is associated with both chronic rejection and graft fibrosis (Jackson et al. 2020; Miyagawa-Hayashino et al. 2012; Tokodai et al. 2018). Post-transplant de novo DSA are associated with a highly mismatched graft and/or with under-immunosuppression (Zhang 2018). According to this concept, increasing immunosuppression would be expected to inhibit graft rejection and thereby attenuate fibrosis in pediatric LT patients. However, the benefit of increasing immunosuppression on graft histological results has not been unequivocally clear. Evans et al. reported that steroid therapy decreased histological hepatitis, but not the degree of fibrosis (Evans et al. 2006; Venturi et al. 2014). In a long-term follow-up trial focused on pediatric LT (Scheenstra et al. 2009), the graft fibrosis was not significantly correlated to either rejection or chronic hepatitis, nor to the presence of a calcineurin inhibitor in the immunosuppressive regimen. Finally, the presence of DSA, as a marker of alloimmunity, has not been confirmed in all studies as a predictor of the development of fibrosis. Vandevoorde et al. described in patients at 10 years post-LT that severe fibrosis was present in 11.1% of DSA-positive and 10.3% of DSA-negative patients (Vandevoorde et al. 2018). Thus, although immune phenomena may play a role in graft fibrosis, its precise function and mechanism, as well as its relative contribution to other factors is still unclear.

As indicated above, prolonged cold and warm ischemia time was reported as risk factor for portal graft fibrosis after pediatric LT (Chanpong et al. 2019; Scheenstra et al. 2009; Venturi et al. 2014). Ischemia–reperfusion injury (IRI) has long been recognized to induce the release of endogenous molecules from apoptotic and necrotic cells, named danger- or death-associated molecular patterns (DAMPs), which may play a role in fibrogenesis (Mihm 2018). However, it is not clear whether this IRI is indeed mechanistically involved in “silent” graft fibrosis detected by protocol biopsies years after transplantation. Similarly, the mechanisms by which high donor age, partial grafts or deceased donor organs specifically contribute to a higher prevalence of graft fibrosis after pediatric LT has not been resolved. (Chanpong et al. 2019; Scheenstra et al. 2009; Venturi et al. 2014). In summary, much of the research up to now has been descriptive in nature, displaying argumentative results and thus the risk factors indicated above still need elucidation of the pathogenic mechanisms.

### Altered microbial composition/function in patients undergoing LT

Pediatric patients with end-stage liver diseases, such as due to biliary atresia or other cholestatic diseases, often have an altered microbial composition (Guo et al. 2018; Wang et al. 2020a; Wang et al. 2019). Such alteration in the microbiome would possibly lead to gut barrier disruption, to bacterial translocation and to triggering of the host’s immune and metabolic responses in the liver (De Minicis et al. 2014; Fouts et al. 2012). For instance, infants with biliary atresia have lower microbial diversity and higher intestinal permeability than healthy infants (Wang et al. 2020a). In particular, patients showed a decrease in the relative abundance of genera which are considered beneficial, i.e., *Bifidobacterium* and *Faecalibacterium* (Guo et al. 2018; Wang et al. 2019), as well as an imbalance in the components of bile acids (Wang et al. 2019). The surgical procedure of LT may temporarily increase the intestinal permeability and allow some pathogenic bacteria to enter the portal or systemic circulation and initiate the immune response (Okumura and Takeda 2018). The administration of prophylactic antibiotics and of immunosuppressants at the perioperative and early post-LT period can also decrease microbial diversity (Kato et al. 2017; Lu et al. 2013) and induce colonization of multidrug-resistance bacteria (Annavajhala et al. 2019), contributing to the risk of post-LT infections. Antibiotic treatment showed various effects on intestinal permeability upon using different antibiotic classes (Tulstrup et al. 2015). Mice with long-term exposure to low doses of penicillin, exhibited accelerated fibrogenesis in response to a high-fat diet compared with control mice, indicating that the combination of antibiotics and a high-fat diet increased liver fibrosis in an NAFLD model (Mahana et al. 2016). Similarly, in mouse models of chronic liver injury, liver fibrosis was more common in germ-free mice than in conventional mice (Mazagova et al. 2015). These data suggested that existence of hepatoprotective microbiota might help prevent or mitigate liver fibrosis in vivo. In human studies, rifaximin, a non-absorbable antibiotic commonly used in advanced liver diseases, may exert beneficial impact by shifting the microbial functionality (Ponziani et al. 2015). One clinical trial of rifaximin has been proposed to assess the effect of gut microbiota on liver fibrosis in humans, however results have not yet been published (Madsen et al. 2018).

### Restoration of altered microbial composition/function by LT

So far, studies on the microbial response to LT are scarce in pediatric patients. Several LT studies in adults have suggested that LT positively impact on the gut microbiome by improving the microbial diversity and composition (Table 1). For instance, Sun et al. assessed fecal microbiome in pre-LT and post-LT fecal samples from 9 LT patients, as well as in 15 healthy controls (Sun et al. 2017). They found that the microbiome in pre-LT patients was significantly different from that in post-LT ones and in healthy controls, while no significant difference observed between the latter two groups. This study provides indications that LT, at least partially, restores the composition of the intestinal microbial community. Compared to pre-LT samples, the post-LT microbiome showed a decrease in the relative abundances of *Actinobacillus*, *Escherichia* and *Shigella*, but an increase in the abundance of *Micromonosporaceae* and *Akkermansia.* Interestingly, *Akkermansia muciniphila* is associated with gut-barrier integrity and the reduction in the abundance of *A. muciniphila* was correlated with thinning of the mucus layer and increased liver inflammation (Everard et al. 2013; Grander et al. 2018; Wu et al. 2017). Similarly, Bajaj et al. (2017, 2018a) also showed that LT increased microbial diversity, decreased potentially pathogenic bacteria taxa, such as the genera belonging to *Enterobacteriaceae*, and increased potentially beneficial taxa, such as *Ruminococcaceae*, along with amelioration in cognition and life quality of patients.

**Table 1:** Summary of studies on gut microbiome in LT recipients

Study	Comparison^a^	Implicated Microbiota		Key observations
		Phylum	Genus	
(Sun et al. 2017)	post-LT vs pre-LT	–	Actinobacillus, Escherichia, Shigella, Anaerolineaceae, Fusobacteriales, Clostridium (sensu stricto), Fusobacteriaceae, Aeromonas, and Clostridium cluster XVIII ↓ Micromonosporaceae, Desulfobacterales, Eubacteriaceae, Sarcina, Akkermansia, Chitinophagaceae, and Coriobacteriaceae ↑	1. Fecal microbial communities were significantly altered following LT. 2. Gut microbiota composition of post-LT patients was more similar to that of healthy controls. 3. The abundance of Akkermansia was higher in all post-LT samples than pre-LT ones.
(Bajaj et al. 2017)	post-LT vs pre-LT	–	Escherichia, Shigella, Salmonella, Bifido bacteriaceae↓ Ruminococcaceae, Lachnospiraceae, Clostridiales Cluster XIV, Streptococcaceae, and Desulfovibrioceae↑	1. There was a significant increase in diversity after LT, while controls had the highest diversity. 2. Post-LT patients have less beneficial microbiota taxa compared to healthy controls, indicating residual dysbiosis.
	post-LT vs healthy	–	Lachnospiraceae, Ruminococcaceae, Clostridiales Cluster XIV, Streptococcaceae, Eubacteriaceae, and Bifidobacteriaceae↓ Bacteroidaceae, Bradyrhizobiaceae, and Veillonellacae↑	
(Bajaj et al. 2018a)	post-LT vs pre-LT	–	Escherichia, Shigella, Salmonella↓ Ruminococcaceae and Lachnospiraceae↑	LT improves gut microbiota diversity and dysbiosis.
(Lu et al. 2019)	post-LT vs healthy	Firmicute↓Bacteroidete↑	–	1. Opportunistic pathogens seemed to be enriched in the liver recipients. 2. The recipients showed less diversity in butyrate-producing bacteria compared with healthy controls.
(Kato et al. 2017)	post-LT vs pre-LT	–	–	The microbial diversity decreased during the first 3 weeks after LT and gradually increased afterwards.

^a^Condition A vs B, ↓ Decrease in A compared to B; ↑ Increase in A compared to B

*LT* liver transplantation

### Contribution of altered microbial composition/function to graft fibrosis

**Fig. 1:**
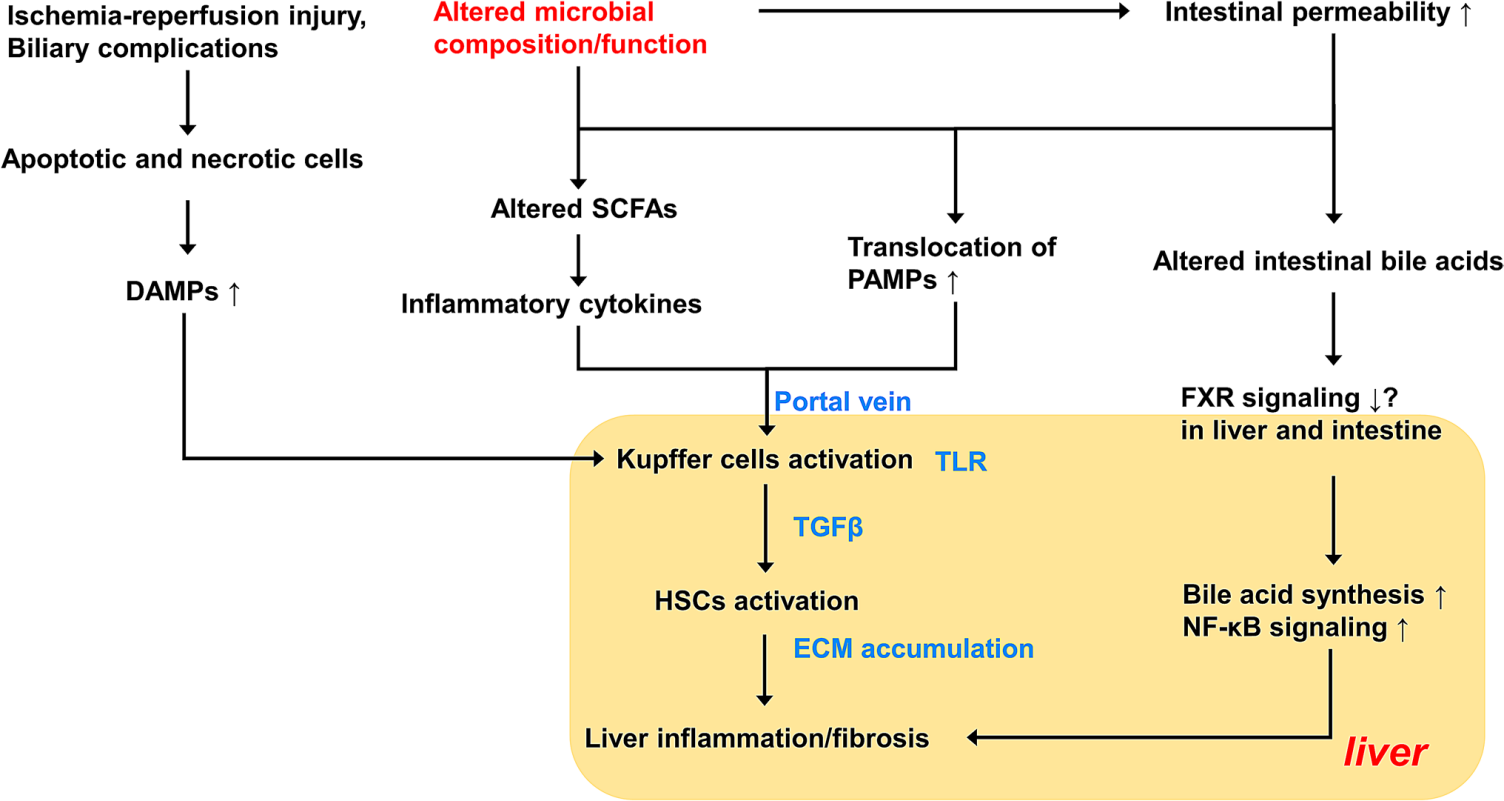
Possible mechanisms of persistent dysbiosis in liver fibrosis. *DAMP* damage-associated molecular pattern, *ECM* extracellular matrix, *FXR* farnesoid X receptor, *PAMP* pathogen-associated molecular pattern, *SCFA* short-chain fatty acid, *TGFβ* transforming growth factor β, *TLR* toll-like receptor

Generally speaking, LT has a positive impact on the gut microbiome (Bajaj et al. 2017, 2018a; Sun et al. 2017) (Table 1). However, some post-LT patients may display persistently altered microbial composition/function, which in turn influences the liver (Bajaj et al. 2017; Lu et al. 2019). Below we discuss several potential underlying mechanisms through which persistently altered microbial composition/function could contribute to the risk of post-LT graft fibrosis (Fig. 1).

#### Translocation of PAMPs due to higher intestinal permeability

**Fig. 2:**
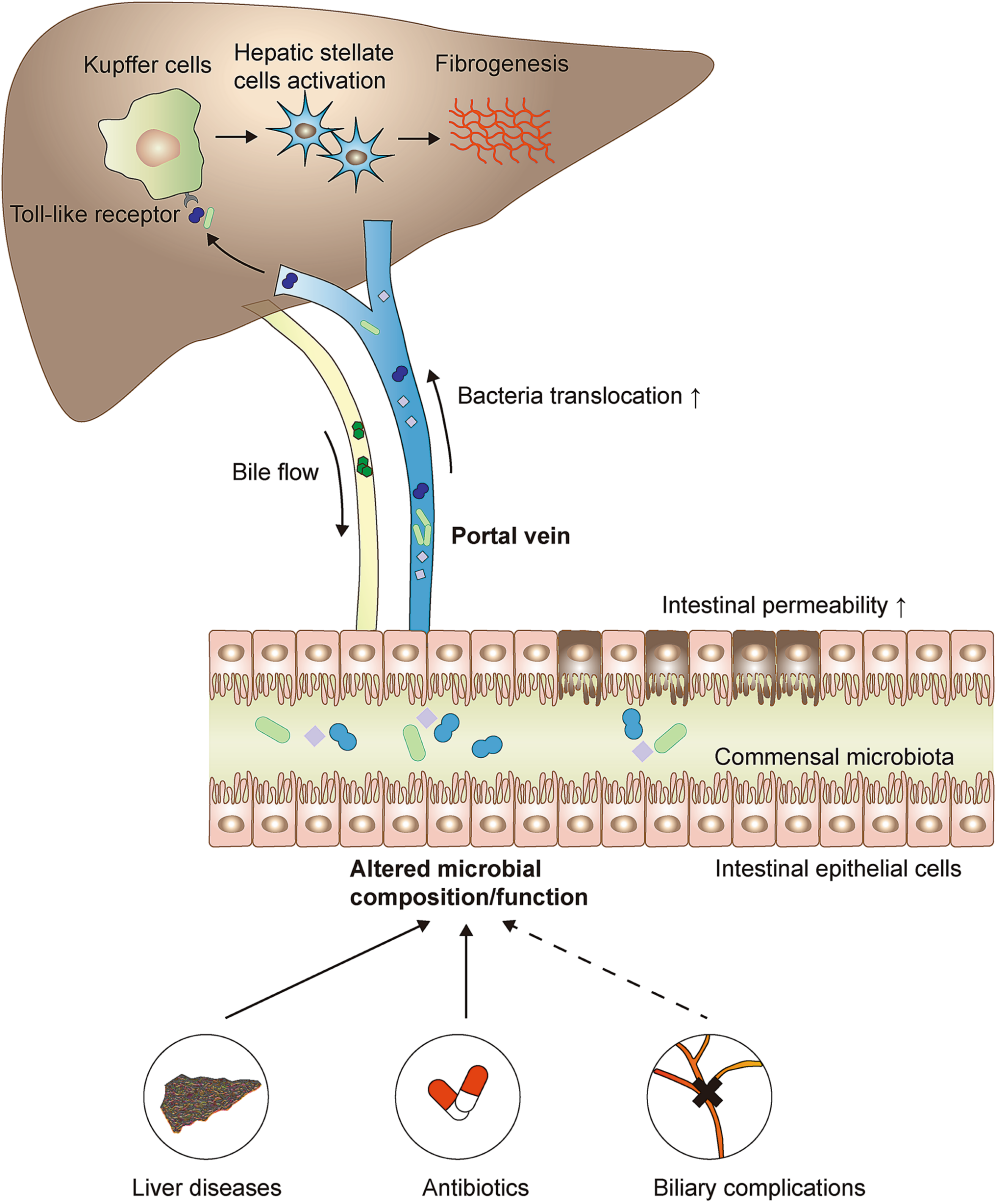
Translocation of PAMPs due to higher intestinal permeability. Alteration of the intestinal microbiome, caused by liver diseases, administration of antibiotics or potential post-LT complications, is hypothesized to contribute to the graft fibrogenesis. Thus, the altered microbial composition/function leads to increased intestinal permeability and translocation of bacteria along with PAMPs to liver via the portal vein. Translocated PAMPs activate TLRs on hepatic Kupffer cells to induce the proinflammatory pathways, further resulting in the activation of HSCs. HSCs produce the ECM and initiate the liver fibrogenesis. *ECM*, extracellular matrix, *HSC* hepatic stellate cell, *PAMP* pathogen-associated molecular pattern, *TLR* toll-like receptor

One mechanism could be that altered microbial composition results in impaired gut barrier integrity, increases the translocation of microbes and microbial products across the gut epithelium, such as pathogen-associated molecular patterns (PAMPs) (Fouts et al. 2012). With increased intestinal permeability, harmful pathogens and/or products originating from the gut lumen travel across the gut barrier and reach the local mesenteric lymph nodes, which are unable to provide an adequate clearance when the amount of translocated PAMPs surpasses their surveillance activity (Albillos et al. 2020; Fouts et al. 2012). In that condition, translocated microbes and their products can translocate to the liver through the portal vein, initiating and aggravating an innate immune activation in the transplanted organ (Seki and Schnabl 2012) (Fig. 2).

PAMPs, i.e., lipopolysaccharide (LPS), microbial DNA, peptidoglycans, and lipopeptides, are then recognized via pattern recognition receptors (PRRs) in the liver (Chen et al. 2019; Seki et al. 2007). Toll-like receptors (TLRs), which are expressed in all cell types in the liver, are the most studied PRRs. The interaction between PAMPs and TLRs elicits host immunological responses via Kupffer cells, either MyD88-dependent or MyD88-independent, resulting in the activation of nuclear factor-kappa B (NF-κB) and the production of inflammatory cytokines and chemokines (Seki et al. 2007). As such, the downstream inflammasome‐mediated pathways (e.g., TGFβ signaling) stimulate the synthesis of ECM by hepatic stellate cells (HSCs), potentially leading to hepatic inflammation and fibrosis. Several TLR deficient mouse strains or cells were protected against liver injury and fibrosis (Gabele et al. 2008; Hartmann et al. 2012; Seki et al. 2001, 2007), supporting the importance of microbiota in mediating liver fibrogenesis.

#### Imbalanced bile acid metabolism

Bile acids form a class of cholesterol-derived amphipathic compounds that circulate between the gut and the liver in the so-called enterohepatic circulation. Bile acids have important physiological roles in dietary lipid absorption, microbiome modulation, metabolism, liver function, and bile production (de Boer et al. 2018). A healthy bile acid metabolism requires both hepatic and microbial metabolism and indeed, an altered microbial composition has been associated with corresponding changes in bile acid level or composition (Sayin et al. 2013; Wang et al. 2020b). Bile acids are the endogenous ligands of farnesoid X receptor (FXR), a nuclear bile acid receptor, which enhances the epithelial barrier integrity (Gadaleta et al. 2011) and provides negative feedback on hepatic de novo bile acids synthesis (Li et al. 2017; Schumacher et al. 2020). Studies showed that FXR agonists exert an anti-fibrotic role in animal models via suppression of NF-κB signaling (Verbeke et al. 2016; Wang et al. 2008). Imbalance of so-called primary (i.e., synthesized by the liver) and secondary (i.e., having undergone structural modifications by the intestinal microbiome) bile acids might exert regulatory effects on the liver inflammatory response and the gut barrier function via FXR signaling. Interestingly, in a recent study, the administration of probiotics to restore intestinal microbial composition could mitigate the liver fibrosis through inhibiting FXR mediated hepatic bile acid synthesis (Liu et al. 2019).

#### Decreased production of short-chain fatty acids (SCFAs)

SCFAs, including acetate, propionate and butyrate, are the main end products after degradation of dietary fiber by gut microbiota and have several beneficial impacts on host health. SCFAs have an important role in maintenance of host intestinal barrier integrity (Wang et al. 2012), the immune system and metabolism (Bach Knudsen et al. 2018; Mörkl et al. 2018; Schulthess et al. 2019). The immune regulatory function of SCFAs has been described in several studies, which involves the activation of NF-ĸB signaling, the production of proinflammatory cytokines and the activity of regulatory T-cells (Tregs) (Arpaia et al. 2013; Smith et al. 2013; Usami et al. 2008). Fecal and circulating SCFAs have immunomodulatory functions, and have been related to type 2 diabetes, inflammatory bowel diseases and NAFLD (Ding et al. 2019; Müller et al. 2019; Parada Venegas et al. 2019). Supplementing dietary of SCFAs or stimulating SCFA producing bacteria via probiotic approaches could have therapeutic effects (Segain et al. 2000; Weitkunat et al. 2017). Microbial dysbiosis could also be characterized by the reduction of SCFA producing microbiota, which is also indicated in adult LT recipients (Lu et al. 2019). If alteration in microbial composition/function was not restored post-LT, several vital functions of SCFAs for the host, including maintaining intestinal barrier integrity and immune regulation, can be disrupted, which could subsequently lead to liver damage.

### Modulation of microbial composition/function to prevent graft fibrosis

The gut microbiome is to some extent a modifiable entity (Caporaso et al. 2011). During early life, children may be more sensitive to changing the composition of the microbiome by diet or other factors, due to the still underdeveloped microbial colonization in pediatric gut (Derrien et al. 2019). Understanding the role of the gut microbiome in the development of graft fibrosis post-LT can open new avenues for the development of microbiome-based biomarkers for early diagnosis and microbiome-targeting approaches for disease prevention. Such approaches include dietary intervention, administration of probiotics/prebiotics, and even fecal microbiota transplantation.

#### Diet recommendation and nutrition advice post LT

Nutritional support is very crucial in children undergoing LT. Infants with end-stage liver diseases often exhibit growth failure due to impaired absorption of nutrition (Yang et al. 2017). LT outcomes improve if malnutrition can be resolved before surgery and a good nutritional status should be maintained post-LT (Yang et al. 2017). In addition, supplementing fiber and probiotics to LT recipients decreased postoperative infections (Rayes et al. 2002). The impact of nutrition on fibrosis could (partly) be mediated through affecting the microbiome composition or function. Dependent on the age of children, fibers (especially oligosaccharides) are important sources of SCFAs and promote the growth of beneficial bacteria. The American Health Foundation recommends that children over 2 years old should take as daily amount of fiber their weight in years +5 to +10 g/d (Catzola and Vajro 2017). In general, supplementing fiber-containing diet post-LT might improve the gut health in infants. It is tempting to speculate that this could also prevent or mitigate graft fibrosis.

#### Probiotics and prebiotics

Probiotics refer to living bacteria that can benefit the health of the host, such as *Lactobacillus* and *Bifidobacterium*. Prebiotics are compounds in diet that induce the growth or activity of beneficial microorganisms. The beneficial role of probiotics and prebiotics in liver diseases has been documented in animal and human studies (Dhiman et al. 2014; Liu et al. 2017; Liu et al. 2019; Shi et al. 2017; Vajro et al. 2011). In murine models of liver fibrosis, administration of prebiotics reduced fibrosis and inflammation by reversing gut dysbiosis, decreasing production of inflammatory cytokines and downregulating expression of fibrogenic genes (Liu et al. 2017; Shi et al. 2017). *Lactobacillus rhamnosus GG*, a specific probiotic strain, prevents bile acid associated liver injury and fibrosis in mice (Liu et al. 2019). In human studies, probiotic supplementation also showed a beneficial effect. Corresponding to animal studies, probiotic treatment with *Lactobacillus rhamnosus GG* in obese children (Vajro et al. 2011) revealed a significant decrease in alanine aminotransferase, irrespective of changes in BMI (body-mass index). In another randomized controlled study, children receiving VSL#3 (a mixture of 8 probiotic strains) once daily for 4 months showed significant improvement in fatty liver disease severity, and a substantial reduction in BMI (Alisi et al. 2014). In transplant-related studies, administration of probiotics and prebiotics has beneficially impacted the liver graft in the short term (Jorgenson et al. 2018; Rayes et al. 2002). Pre-LT probiotic/prebiotic use reduced the postoperative infection rate as well as the length of hospitalization and of antibiotic use, based on a meta-analysis of four controlled studies (Sawas et al. 2015). Early biochemical tests of graft function improved although the long-term outcome appeared not different (Grat et al. 2017). Administration of probiotics in mouse LT model helped alleviate the acute rejection after surgery by improving the immune parameters, such as Treg cells (Xie et al. 2014). Probiotic/prebiotic administration warrants consideration as a therapeutic tool to treat gut dysbiosis with minimal side effects, and to reconstruct a healthy microbiome community, possibly alleviating liver injury and fibrosis.

#### Fecal microbiome transplant (FMT)

FMT is emerging as a powerful therapeutic approach for the treatment of *C. difficile* infection (Smits et al. 2016), as well as in patients with solid organ transplants (Cheng et al. 2019b; Kelly et al. 2014; Lin et al. 2018a; Shogbesan et al. 2018). LT recipients are more susceptible to *C. difficile* due to immunosuppression therapy, antibiotic treatment, and prolonged hospitalizations (Lin et al. 2018b). The efficacy and safety of FMT treatment have been proven in several LT cases (Lin et al. 2018b; Schneider et al. 2018). Notably, a recent multicenter study suggested repeated FMT with/without antibiotics therapy in LT recipients would improve the cure rate comparable to immunocompetent patients (Cheng et al. 2019a). Moreover, FMT has also been implemented for the treatment of metabolic disorders, like type 2 diabetes (Vrieze et al. 2012), NAFLD (Yuan et al. 2019), and decompensated cirrhosis (Bajaj et al. 2018b). This is based on the evidence that the restoration of the gut microbiome via FMT can impact on host’s metabolism. In a phase 1 clinical trial, decompensated cirrhosis patients received 5 days of broad-spectrum antibiotics followed by FMT from a donor enriched in *Lachnospiraceae* and *Ruminococcaceae* (Bajaj et al. 2018b). In 15 days, FMT has restored the antibiotic-associated disruption of microbiota composition and improved fecal SCFA and BA profile. Thus we postulate that the simultaneous or consecutive transplantation of a healthy liver and healthy gut microbiome in patients with end-stage liver disease can reduce the risk of infection and help re-establish the healthy gut-liver axis, thereby increasing the prognosis rate of the liver graft.

### Experimental framework to study the microbial role in fibrosis

**Fig. 3:**
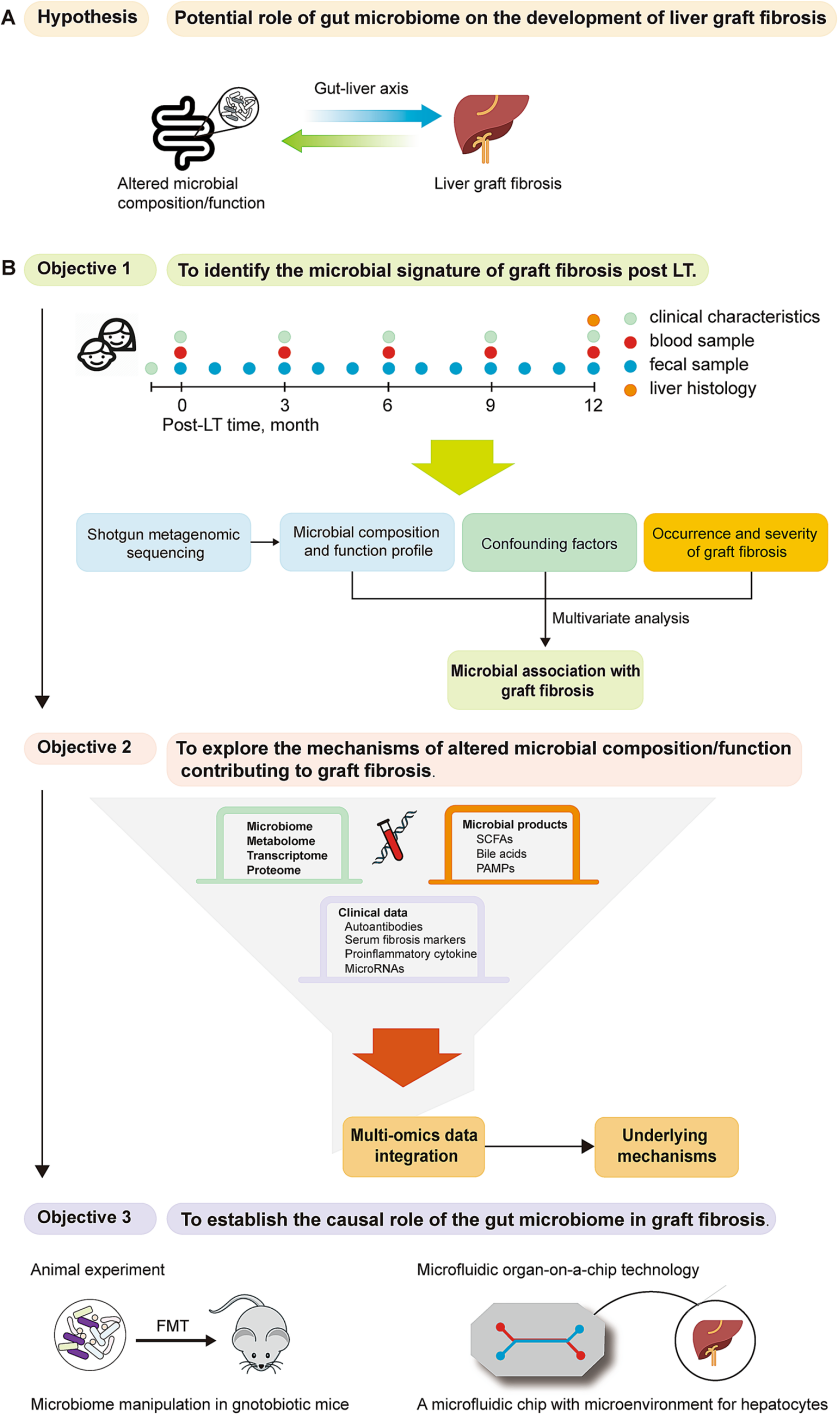
Experimental framework to understand the role of microbial composition/function on the development of graft fibrosis post LT. **a** Hypothesis that altered gut microbial composition/function contributes to the development of graft fibrosis in pediatric LT; **b** Experimental framework containing three objectives to systematically evaluate various aspects of the hypothesized role of the gut microbiome on graft fibrosis after pediatric LT. *FMT* fecal microbiota transplant, *LT* liver transplantation, *PAMP* pathogen-associated molecular pattern; *SCFA* short-chain fatty acid

As indicated above, the alteration of gut microbiome composition/function can be an important determinant in graft fibrosis after pediatric LT (Fig. 3a). Despite the high potential of microbiota-modulating approaches in preventing or mitigating graft fibrosis post LT, the supporting observations obtained so far have all been indirect. To date, there is no study evaluating the role of gut microbiota in the development of graft fibrosis in pediatric LT. To assess the hypothesis in a targeted study and, if positive, move towards clinical applications, we propose an experimental framework that contains three specific objectives to systematically determine various aspects of the intestinal microbiota on graft fibrosis (Fig. 3b).

#### Objective 1: a longitudinal human study in pediatric liver transplant patients to identify the microbial signature of graft fibrosis post LT

The prevalence of graft fibrosis is ~ 50% at 1 year after pediatric LT. To identify an early microbial signature of graft fibrosis, it is essential to have a longitudinal study up to at least 1 year after pediatric LT, including a protocol liver biopsy for detailed analysis of liver histology and graft fibrosis. Detailed patient’s characteristics should be recorded before and after LT, such as age, gender, weight, diet, and drug usage. Blood samples can be collected at baseline, as well as every 3 months after LT, following a regular standardized protocol. Fecal samples can be collected more frequently, for instance at baseline and monthly after LT. There are two commonly used sequencing technologies to determine the gut microbial composition: 16 s rRNA sequencing and shotgun metagenomic sequencing. 16 s rRNA sequencing is also known as amplicon sequencing, in which a specific, variable region of 16 s rRNA gene (e.g., V1, V3 or V4 region) is amplified and then subjected to sequencing (Weinstock 2012). The shotgun metagenomic sequencing refers to whole genome-wide sequencing, while bacterial genomes are fragmented to small species for sequencing. The use of 16 s rRNA sequencing has been approved to be an efficient and cost-effective strategy for microbial profiling. However, metagenomics sequencing clearly has several advantages over 16 s rRNA sequencing (Malla et al. 2019; Tessler et al. 2017). Firstly, 16 s rRNA sequencing can only identify bacteria, generally up to the genus level. Metagenomic sequencing can identify all kinds of microorganisms at the species and even strain level, including bacteria, viruses, and fungi. Secondly, metagenomic sequencing identifies the abundance of bacterial genes that can directly refer to bacterial functionality, which information cannot be directly obtained by 16 s rRNA sequencing. Thirdly, metagenomics sequencing offers us an opportunity to identify unknown organisms via de novo assembling, which is impossible for 16 s rRNA sequencing. After metagenomic sequencing, various analysis tools, such as MetaPhlan and Humman2 (Franzosa et al. 2018; Segata et al. 2012), can be employed to identify the abundance of different bacterial species and their metabolic pathways. This approach allows us to characterize the diversity of the microbial community. In such a way, the change of microbial compositions and their functional profile after LT can be monitored and it can be assessed to what extent the gut microbiome at baseline and its changes after transplantation can be associated with the occurrence and severity of graft fibrosis. Notably, both the development of graft fibrosis and the gut microbiome are complex. The possible confounding effects from other factors, such as diet and drug usage, need to be taken into account in assessing the microbial association with the development of graft fibrosis using multivariate analysis.

#### Objective 2: multi-omics integration to gain insight into the underlying mechanisms

Once the microbial association with graft fibrosis is established in objective 1, the next logical step is to understand through which mechanistic routes the (persistent or emerging) altered microbial composition/function contributes to the development of graft fibrosis. Multi-omics combined with systemic biology approaches have been considered to be a powerful approach to decipher the underlying molecular basis (Hasin et al. 2017). Firstly, it is important to identify microbial products that can impact the host’s immunity and metabolism, such as SCFAs, bile acids and PAMPs that were discussed above. It needs to be assessed whether graft fibrosis-associated microbial alterations are also associated with abnormal levels of these microbial products. Secondly, it then needs to be understood how these microbial products can affect the host, thereby contributing to the development of graft fibrosis. This requires deep omics profiling in LT patients, including transcriptomics, proteomics, and metabolomics, in addition to the profiling of previously established fibrosis risk factors and biomarkers, such as autoantibodies (Venturi et al. 2014), serum fibrosis markers (e.g., hyaluronic acid, alpha-smooth muscle actin, tissue inhibitor of matrix metalloproteinase) (Varma et al. 2017; Voutilainen et al. 2017), proinflammatory cytokines and microRNAs (Kelly et al. 2016). A historical approach is needed to identify all downstream molecular factors that can be affected by the gut microbiome, followed by pathway and network analysis to converge these factors into molecular pathways. Unfortunately, multi-omics integration often encounters technical challenges related to statistical methods and power issues (Hasin et al. 2017; Misra et al. 2018). Such challenges are even more severe as cross-kingdom omics integration has been recently proposed to understand host-microbe interactions (Chen et al. 2018), in which host omics data are integrated with metagenome-based omics data, namely meta-transcriptomics, meta-proteomics, and meta-metabolomics. Such an analysis would definitely need a huge sample size to ensure satisfying analysis power. This highlights the importance of biobanks and large human cohorts in the big data era.

#### Objective 3: moving from associations to causality and clinical applications

The last step is to address the postulated causal role of the gut microbiome in graft fibrosis. Mouse models can be a useful tool to investigate causality, by observing the phenotypic consequences of microbiome manipulation in gnotobiotic mice (Kubelkova et al. 2016). Several experimental mouse models have been developed to study liver fibrosis (Yanguas et al. 2016). However, when we use mouse models to understand host-microbe interactions in humans, the differences between human and mouse need to be considered. For instance, bile acids profiles are remarkably different between human and mouse, due to the murine-specific *cyp2c70* gene that produces α-muricholic acid (MCA) (de Boer et al. 2018). Recently, a *cyp2c70* knock-out mouse model has been developed, with a humanized bile acid profile (de Boer et al. 2020). Such a model, particularly when germ-free, would be appropriate to investigate the role of the gut microbiome in liver fibrosis via the mechanistic route of bile acids. Nevertheless, it is well known that mouse models frequently fall short in predicting human physiology. In recent years, microfluidic organ-on-a-chip (OoC) technology has been emerging as an innovative, animal alternative method to study human physiology and disease mechanisms (Sun et al. 2019). This technology allows to engineer a microfluidic chip and to create a microenvironment for human cells so that they can behave as they do inside a human body, thereby recapitulating the physiology of a specific organ. Liver-on-a-chip has been employed to identify human-specific drug toxicity (Jang et al. 2019). Another major advantage of the OoC technology is the possibility to employ human genetics into disease etiology, by combining OoC with human pluripotent induced stem cell technology (Rowe and Daley 2019; Workman et al. 2018). With the advance in bacterial culture technique (Lagier et al. 2018), these cutting-edge technologies offer us an opportunity to understand the host-microbe interactions in human disease, including graft fibrosis. Such knowledge is badly needed for developing microbiome-targeting approaches to prevent or at least mitigate the development of graft fibrosis and improve the wellbeing of patients.

## Conclusions

Pediatric LT is a life-saving option for children with end-stage liver diseases. However, about of 50% of patients develop graft fibrosis within 1 year after transplantation. Enormous efforts have been made to find ways to prevent or mitigate liver graft fibrosis, unfortunately, so far without much success. In recent years, accumulating observations suggest that the altered microbial composition/function is an important player in liver diseases and possibly is related to graft fibrosis after pediatric LT. Here we provide a prospective view on the role of the gut microbiome in graft fibrogenesis after pediatric LT and highlight the potential of microbiome-based approaches for early diagnosis, prevention and treatment. To realize this potential, collective efforts from clinical doctors, bioinformaticians, molecular biologists and microbiologists are required to test the hypothesis and, if positive, to obtain direct evidence and to uncover the underlying mechanisms.

## Abbreviations

ALD: Alcoholic liver disease
BMI: Body-mass index
DAMP: Damage-associated molecular pattern
DSA: Donor-specific antibody
ECM: Extracellular matrix
FMT: Fecal microbiota transplant
FXR: Farnesoid X receptor
HSC: Hepatic stellate cell
IRI: Ischemia–reperfusion injury
LT: Liver transplantation
LPS: Lipopolysaccharide
NAFLD: Non-alcoholic fatty liver disease
NF-κB: Nuclear factor kappa B
PAMP: Pathogen-associated molecular pattern
PRR: Pattern recognition receptor
PTLD: Post-transplantation lymphoproliferative disease
SCFA: Short-chain fatty acid
TGFβ: Transforming growth factor β
TLR: Toll-like receptor
